# Mechanical Force in T Cell Receptor Signal Initiation

**DOI:** 10.3389/fimmu.2012.00217

**Published:** 2012-07-20

**Authors:** Zhengyu Ma, Dennis E. Discher, Terri H. Finkel

**Affiliations:** ^1^Department of Pediatrics, Nemours Children's HospitalOrlando, FL, USA; ^2^Department of Chemical and Biomolecular Engineering, University of PennsylvaniaPhiladelphia, PA, USA

In peripheral lymphoid organs, such as lymph nodes, T lymphocytes (T cells) actively scan the surface of antigen presenting cells (APCs) for evidence of pathogen invasion. Proteins of pathogens taken up by APCs are processed to short peptides and presented on the cell surface with major histocompatibility complexes (MHCs). Specific binding between peptide-MHC complexes (pMHCs) on APCs and T cell receptors (TCRs) on T cells initiate signaling cascades from TCRs that eventually lead to T cell activation and proliferation. Despite its importance, how pMHC-TCR binding triggers a signal from the TCR remains to be elucidated (van der Merwe and Dushek, 2011). The central question is: how does pMHC binding change the TCR/CD3 complex to a state that facilitates signaling events such as tyrosine phosphorylation of the ITAM domains of CD3?

The structures of pMHC and TCR molecules and the way they interact have been intensively studied. Detailed structures of dozens of pMHC and TCR molecules, in unbound and bound states, have been solved with atomic-level resolution (Rudolph et al., 2006). The configurational relationships among the eight chains of TCR/CD3 complex and with the plasma membrane have also been dissected using biochemical and biophysical approaches (Kuhns et al., 2006; Xu et al., 2008). Moreover, the steady state, kinetic, and thermal dynamic parameters of the pMHC-TCR binding have been characterized in great detail using methods such as surface plasmon resonance (SPR; Krogsgaard et al., 2003). These advances, however, have not led to a working model that satisfactorily explains the mechanism of TCR triggering.

A factor *often* overlooked is that the behavior of pMHC and TCR may be significantly altered by mechanical forces at the real T cell-APC interface. The interaction between the T cell and APC, especially in a three-dimensional (3D) environment, is highly dynamic (Gunzer et al., 2000; Mempel et al., 2004; Miller et al., 2004): the interaction is perhaps better described as “dancing” rather than “binding.” Relative motions between the two plasma membranes at the interface, in the form of sliding, and/or detaching, are likely frequent. These should inevitably apply mechanical forces on all ligand-receptor interactions at the interface, including between pMHC and TCR. In addition, bound pMHC and TCR may be mechanically stressed by nearby larger molecules that push membranes apart like springs (Shaw and Dustin, 1997; Li et al., 2010; Allard et al., 2012). Experimental data have come recently in studies of pMHC-TCR stability at the T cell-APC interface. The two-dimensional (2D) dissociation rate of pMHC-TCR interaction measured in a flow chamber-based assay was similar to their intrinsic 3D dissociation rate (Robert et al., 2012). In other studies using mechanical (Huang et al., 2010) and optical (Huppa et al., 2010) methods, however, 2D dissociation rates were found up to 8,300-fold and 12-fold faster than 3D dissociation rates, respectively. In the simplest processes, the dissociation rate increases exponentially with the magnitude of a disengagement force (Bell, 1978). Therefore, accelerated dissociation would indicate that pMHC-TCR interaction at the T cell-APC interface is under mechanical stress.

We and others have begun to consider that mechanical forces drive changes in TCR/CD3 and trigger TCR signaling (Lanzavecchia et al., 1999; van der Merwe, 2001; Ma et al., 2008a,b; Ma and Finkel, 2010). pMHC-TCR binding, in this case, only serves to transmit such forces. In this sense, TCRs behave like molecular sensors in mechanotransduction, translating mechanical forces into biochemical signals (Vogel, 2006). There are at least two ways that this translation could conceivably take place. First, forces exerted on TCR could change CD3 intracellular domains to a conformation or configuration that favors signaling (Gil et al., 2002; Xu et al., 2008; Zhang et al., 2011). For example, mechanical forces could deform the TCR and this conformational change is then transferred to CD3 through interactions between extracellular domains (Kuhns et al., 2006). Alternatively, if the structure of TCR is rigid, forces may be transmitted down to the plasma membrane and directly change the conformation of CD3 domains, as seen with other Ig domain proteins such as VCAM (Bhasin et al., 2004), or else change relative positioning through interactions among their transmembrane domains. Second, if forces are exerted on the TCR horizontally, TCRs could be “dragged” across the T cell surface and collide with membrane domains enriched in tyrosine kinases, such as Lck-rich lipid rafts (Lanzavecchia et al., 1999).

Recent experimental evidence supports the role of mechanical force in TCR triggering. Using pMHC-coated beads manipulated by optical tweezers, Reinherz and colleagues demonstrated that T cell calcium flux is induced by applying a 50 pN force (Kim et al., 2009). Interestingly, the calcium response was seen only when force was applied tangentially relative to the T cell by “rubbing” the beads against the T cell surface. The authors suggested that TCRs work as anisotropic (direction-dependent) mechanosensors and postulated that while a T cell is crawling on the surface of an APC, a pMHC bound to a specific TCR may bend the TCR and push it against the membrane distal lobes of CD3ε. Alternative explanations to the experimental data, however, may exist. The pMHC ligands were coated on a rigid bead surface, probably at high densities. Therefore, T cell calcium flux could have been a result of TCR crosslinking by pMHCs. Also, on the T cell surface, TCRs (which are relatively small) are shielded from easy access to pMHCs anchored on an opposing surface (van der Merwe and Davis, 2003) by a thick layer of large glycoprotein. Tangential movements of pMHC-coated beads may simply work better than vertical movements to push away these glycoproteins, exposing TCRs, and enhancing the chance of pMHC-TCR interaction. In addition, for a tangential force to bend a TCR, the TCR must be relatively fixed on the surface so that it does not easily move laterally when a force is applied. The fluidity of the lipid membrane makes this last mechanism unlikely. Therefore, a tangential force may have induced a calcium response by dragging TCR/CD3 complexes to collide with each other or with Lck-rich lipid rafts (Lanzavecchia et al., 1999).

In a separate study (Li et al., 2010), a single-chain antibody against CD3 (CD3L) linked on top of two Ig domains (CD3L-2d) or to the large extracellular domain of CD43 (CD3L-CD43) was expressed on the surface of 3T3 fibroblasts. CD3L-2d induced robust T cell proliferation and calcium responses, while CD3L-CD43 failed to induce any response. Calcium responses, however, were detected in T cells interacting with 3T3 cells expressing CD3L-CD43 when a shear force was applied using a stream of medium blown from a micropipette, and when a vertical pulling force was applied by pulling the T cells up and away from the 3T3 cells with a micropipette. In contrast to the study using optical tweezers above, these results suggest that TCRs are not sensitive to the direction of force, since both shear force and pulling force triggered signaling. In this study, mechanical forces should not have worked simply by increasing the chance of CD3L-CD3 interaction, since the larger CD3L-CD43 should have better access to CD3 than the smaller CD3L-2d but could not trigger the TCR without external force. This study might have provided more direct evidence for the role of force in TCR triggering, however, if an anti-TCR antibody had been used instead of the anti-CD3 antibody.

To induce certain changes in the TCR/CD3 complex, the magnitude of force is just as important as the direction of force discussed above. T cells can obviously generate a large amount of force to rupture all molecular interactions with the extracellular matrix or adjacent cells while migrating. T cells can even bore holes into endothelial cells when they migrate through (Carman et al., 2007). At least in the case of a pulling force, however, the amount of force exerted on the TCR depends on the mechanical strength of the pMHC-TCR binding. According to the single barrier model for unbinding of ligand and receptors by force (Bell, 1978; Evans and Ritchie, 1997), the most probable unbinding force (*F*) is logarithmically dependent on the intrinsic stability of the binding, as well as the rate of force application, as described by:

$$F=\frac{k_{\text{B}}T}{x}\cdot \ln\left(\frac{rx}{k_{\text{off}}k_{\text{B}}T}\right)$$

where *k*_B_ is the Boltzmann constant, *T* is the absolute temperature, *k*_off_ is the intrinsic off-rate of binding, *x* is the distance from the energy minimum of bound state to activation barrier, and *r* represents the loading rate. When a force is applied through a spring-like object such as the cantilever of an AFM or the membrane of a cell, the loading rate (N/s) equals the object's moving velocity (m/s) multiplied by the object's spring constant (N/m). Therefore, for a given pMHC-TCR interaction with a particular *k*_off_, more force will be delivered to the TCR before the interaction ruptures if either the membrane detachment speed is higher or if the membranes are more rigid.

Most recently, studies have been carried out to characterize the mechanical strength of pMHC-TCR binding under controlled loading rates using AFM. Bozna et al. (2011) measured the force needed to unbind the invariant natural killer T cell receptor (iNKTCR) and the MHC class I-like CD1d molecule loaded with lipid antigens. Two lipid antigens, αGalCer and OCH12, with respective intrinsic iNKTCR binding off-rates of 0.39 and 1 s^−1^, were tested at different force loading rates. At a loading rate of 1 nN/s, the unbinding forces for αGalCer and OCH12 were about 40 and 30 pN, respectively. At a loading rate of 0.1 nN/s, the unbinding forces dropped to about 25 and 15 pN, respectively. Another study by Puech et al. (2011) characterized the interaction between BM3.3 TCR on a T cell hybridoma and H-2Kb presenting BM1 peptide on an AFM tip. A very short tip-cell contact time of ∼300 ms was followed by a pulling force with a large loading rate of 10 nN/s. A small unbinding force of about 20 pN was measured, and the magnitude was independent of the presence of specific BM1 peptide. The authors reasoned that this was because pMHC-TCR interactions were ruptured before the bond was fully matured due to the very short contact time.

The magnitude of force in the tens of pN range as measured in the above studies may be sufficient to induce meaningful changes in the TCR/CD3 complex that lead to TCR signaling. A force of 12 pN was sufficient to stretch the intracellular domain of talin and expose cryptic binding sites for the binding of vinculin (del Rio et al., 2009). Additional membrane cytoskeletal proteins that undergo conformational changes at similar forces when stressed by AFM have also been found to unfold in intact cell membranes under stress as detected by mass spectrometry methods (Johnson et al., 2007). The magnitude of force sustained by TCR at the real T cell-APC interface, however, remains to be determined. Although the intrinsic off-rate of pMHC-TCR binding can be determined by SPR, the loading rate(s) for forces at the T cell-APC interface are unknown and likely to vary greatly depending on local membrane separation speed and local membrane rigidity. Recent studies have begun to investigate this issue. Using a biomembrane force probe (BFP), Husson et al. (2011) measured the forces naturally generated by a T cell interacting with a bead coated with anti-LFA-1 or anti-CD3 antibodies. Anti-LFA-1 beads triggered only a small force with a very slow loading rate of 0.2 pN/s. Anti-CD3 beads, however, induced a complex, three-phase force response: latency phase, pushing phase, and pulling phase. No detectable force was measured during the latency phase. This was followed by a strong pushing phase, then a stronger pulling phase with a loading rate of 2 pN/s. If loading rates at the T cell-APC interface are indeed in the range of a few pN/s, the forces sustained by pMHC TCR binding should be much smaller than what was measured in the AFM studies above. It should be noted, however, that the experimental setup used plastic beads interacting with an isolated T cell, which does not realistically mimic the complex interaction between a T cell and an APC. Further studies under more physiological settings are necessary.

In summary, mechanical force is likely to be an integral part of T cell-APC physiology and key to the mechanism of TCR triggering by pMHC. Mechanical forces leading to receptor deformation seems to be the only model that can address all three aspects of the T cell triggering puzzle: mechanism, specificity, and sensitivity (Ma et al., 2008a). New experimental evidence in support of the role of force in TCR triggering has started to emerge. Recent advances in AFM and optical tweezers should allow in-depth analysis of mechanical forces at the single-molecule level at the T cell-APC interface. New insights might then answer the fundamental question of how T cells distinguish what is foreign and what is self.
